# Supplementary material for: The genome sequence of a cased caddisfly, Ceraclea dissimilis (Stephens, 1836)
Source: Wellcome Open Res. 2025 Sep 26;9:308. Originally published 2024 Jun 11. [Version 2] doi: 10.12688/wellcomeopenres.22268.2 (PMC12635522; doi:10.12688/wellcomeopenres.22268.2)
Supplement: Peer review history

# Reviewer report: Reviewer response for version 2

**DOI:** 10.21956/wellcomeopenres.27560.r134717

**Published:** 2025-11-20

**Authors:** John S. Sproul

**Affiliations:** 1 Brigham Young University, Provo, Utah, USA

In this article the authors sequenced and assembled the nuclear and mitochondrial genome sequences of ceraclea dissimilis, a caddisfly in the family Leptoceridae. The authors used Pacific Biosciences HiFi sequencing for primary data generation and complemented their initial assembly using Hi-C scaffolding. The authors checked for contamination and evaluated assembly quality using blobtools, BUSCO, and Mercury.FK. The resulting assembly appears to be of excellent quality, with the large majority (>99%) of sequences being assigned to putatively resolved chromosomes. 

 The work appears to meet high standards of sequencing and initial analysis. The genomic resource might be improved with annotations of genes or repetitive elements; however, the assembly will be of use as-is to the biodiversity genomics community.

Are sufficient details of methods and materials provided to allow replication by others?

Yes

Is the rationale for creating the dataset(s) clearly described?

Yes

Are the datasets clearly presented in a useable and accessible format?

Yes

Are the protocols appropriate and is the work technically sound?

Yes

Reviewer Expertise:

Evolutionary biology, biodiversity genomics of repetitive elements, entomology, Carabidae

I confirm that I have read this submission and believe that I have an appropriate level of expertise to confirm that it is of an acceptable scientific standard.

# Reviewer report: Reviewer response for version 1

**DOI:** 10.21956/wellcomeopenres.24545.r89266

**Published:** 2024-08-14

**Authors:** Terrence Sylvester

**Affiliations:** 1 The University of Memphis, Memphis, Tennessee, USA

The authors present a high-quality genome sequence of *Ceraclea dissimilis*. The author provides sufficient background on the caddisfly and clearly describes the rationale for creating the dataset. The manuscript follows a standard, widely accepted genome sequencing and assembly approach. The authors provide sufficient details on methods (including the programs used and versions) for replication of this study by others. Data are provided in a usable and accessible format. The assembly itself is of excellent quality however can further benefit from having an annotation. I do not have significant remarks on the work presented in this manuscript.

Are sufficient details of methods and materials provided to allow replication by others?

Yes

Is the rationale for creating the dataset(s) clearly described?

Yes

Are the datasets clearly presented in a useable and accessible format?

Yes

Are the protocols appropriate and is the work technically sound?

Yes

Reviewer Expertise:

genomics

I confirm that I have read this submission and believe that I have an appropriate level of expertise to confirm that it is of an acceptable scientific standard.

# Reviewer report: Reviewer response for version 1

**DOI:** 10.21956/wellcomeopenres.24545.r89272

**Published:** 2024-08-02

**Authors:** Manee M Manee

**Affiliations:** 1 King Abdulaziz City for Science and Technology, Riyadh, Saudi Arabia

The study provides a high-quality, chromosomal-level genome sequence for *Ceraclea dissimilis*, contributing valuable genomic data for the species. Utilization of Pacific Biosciences HiFi long reads and Hi-C data to achieve comprehensive and accurate genome assembly. While the genome assembly is nearly complete based on the BUSCO score, the study lacks detailed functional annotation of genes and regulatory elements.

Are sufficient details of methods and materials provided to allow replication by others?

Yes

Is the rationale for creating the dataset(s) clearly described?

Yes

Are the datasets clearly presented in a useable and accessible format?

Yes

Are the protocols appropriate and is the work technically sound?

Yes

Reviewer Expertise:

bioinformatics, genomics and evolutionary biology

I confirm that I have read this submission and believe that I have an appropriate level of expertise to confirm that it is of an acceptable scientific standard, however I have significant reservations, as outlined above.

# Response

**Published:** 2025-09-22

**Authors:** Tree of Life Team Sanger

**Affiliations:** Wellcome Trust Sanger Institute, UK

Thank you for reviewing this data note. 

 We acknowledge the comment regarding genome annotation. At present, this genome has not been annotated. The purpose of this data note is to describe and evaluate the assembly itself, which can and should be assessed on its own merits. Annotation is a separate downstream step, performed externally by Ensembl at the European Bioinformatics Institute, and lies outside the scope of this study.
